# Community health worker perspectives on advocacy: design-based research to develop a digital advocacy training course

**DOI:** 10.3389/fpubh.2024.1334279

**Published:** 2024-04-10

**Authors:** Nophiwe Job, Jamie Sewan Johnston, Carey Westgate, Nadine Ann Skinner, Victoria Ward, Madeleine Ballard, CHW Advisory Group

**Affiliations:** ^1^Stanford Center for Health Education, Cape Town, South Africa; ^2^Stanford Center for Health Education, Stanford, CA, United States; ^3^Community Health Impact Coalition, London, United Kingdom; ^4^Stanford University School of Medicine, Stanford, CA, United States; ^5^Icahn School of Medicine at Mount Sinai, New York, NY, United States

**Keywords:** CHWs, health advocacy, advocacy training, design-based research, digital training

## Abstract

**Introduction:**

While community health workers (CHWs) are well-positioned as health advocates, they frequently lack support and feel undervalued. Advocacy training may prepare CHWs to support communities better.

**Methods:**

This study uses a design-based research approach to (1) explore how participation in curriculum-development workshops for a digital advocacy course influenced CHWs’ (*n* = 25) perceptions of advocacy and (2) describe how CHW involvement shaped course development. Data were collected via five discussion groups and seven surveys over six months.

**Results:**

Initially, the CHWs perceived themselves as community-advocates but not as self-advocates. They increasingly reflected on the merits of advocating for better working conditions and aspired to greater involvement in decision-making. CHWs reflected positively on their advisory role in shaping the course to improve content acceptability and validity.

**Discussion:**

Training efforts to engage CHWs in advocacy must overcome systemic barriers and norms internalized by CHWs that deter them from reaching their full potential as advocates.

## Introduction

Community health workers (CHWs) are well-positioned as health advocates due to their unique roles, relationships, and insights into the issues their communities face (1–4). Successful health advocates can effectively mobilize their communities and improve public health outcomes (5–8). CHWs and other frontline health workers trained in community advocacy report improved organizational trust (9) and a greater understanding of their role as health agents (10). These trained advocates have the potential to address critical structural health issues such as poverty, employment, housing, and discrimination (1).

While CHWs play a crucial role in public health systems, particularly in low-and middle-income countries (LMICs), CHW programs frequently lack sufficient planning, support, training, supervision, and resources (11). Lack of institutional support, including limited supervision, insufficient financial remuneration, and inadequate supplies, equipment, and training, as well as CHWs feeling undervalued and underappreciated in their efforts by healthcare colleagues, often leads CHWs to express feelings of powerlessness over their work and work environment and to perceive that they are not valued members of the healthcare system (11–18). In some low-resourced areas, entire CHW programs have failed due to the high attrition associated with the lack of support that CHWs have received (11, 19, 20). Even if programs continue, the ongoing failure to support CHWs can limit their ability to provide patient health services and decrease vulnerable communities’ trust in healthcare systems (12).

Although CHWs are well-positioned as community health advocates, more evidence is needed to understand how to foster and encourage advocacy among CHWs. Evidence suggests that advocacy and leadership training for CHWs improves the odds of CHWs engaging in political, civic, and workplace advocacy (1, 9, 21–24) and creating cohorts of CHWs trained in advocacy (1). CHWs receiving advocacy training may be better positioned to address issues impacting their profession and their patients. Nevertheless, few studies have examined the role of including CHWs in the development of advocacy training for CHWs in LMICs.

The Community Health Impact Coalition (CHIC) is a network of CHWs and aligned health organizations in over 40 countries making professional CHWs the norm worldwide by changing guidelines, funding, and policy. CHIC partnered with the Stanford Center for Health Education’s Digital Medic initiative to develop a digital advocacy training course through a design-based research (DBR) approach. Originating from critiques that education research can fail to consider context and yield results too abstract to be applied in real-world education settings, the DBR methodological approach involves research subjects as collaborators to help formulate, refine, and evaluate educational interventions (25–31). In the present study, CHWs played a key role as advisors in course development: providing iterative feedback on the course structure, course learning objectives, prototype scripts and visuals and evaluating the effectiveness of the course content.

Using a purposive sample of 25 CHWs from eight countries, this study explores how participation in a series of advocacy training curriculum-development workshops influenced CHWs’ perceptions of their role as advocates for their profession and for their patient communities. Specifically, we examine participants’ (1) awareness of the role CHWs play in health systems globally, (2) confidence and motivation to engage in advocacy activities, and (3) sense of connectedness with CHWs from different countries and regions to support one another through shared knowledge, advocacy, and health practices. We also examine how CHW participants perceived their role as advisors in the course development process and describe how their participation influenced the course content and instructional design.

## Background

In 2020, CHIC launched the CHW Advocates Campaign to include CHWs in high-level decision-making by ensuring ‘nothing about CHWs without CHWs’. As part of the CHW Advocates Campaign, CHIC and Digital Medic collaborated to create a digital advocacy training course to support CHWs to develop their leadership and advocacy skills. This study employs the World Health Organization’s (WHO) definition of advocacy to understand CHWs experience with this course. The WHO’s definition is “advocacy for development is a combination of social actions designed to gain political commitment, policy support, social acceptance, and systems support for a particular goal or programme” (32). The CHW Advocacy Training course^1^ aims to create a network of CHWs trained to participate and successfully advocate for the needs of their profession and their patient communities. The course curriculum draws upon advocacy training for healthcare providers and is organized into four key modules.

The first module of the course focuses on the collective identity of CHWs to help CHWs create a shared professional identity and consciousness. Research on advocacy has indicated that one of the primary pre-conditions for advocacy or movement development is that certain members of society identify as a collective group that feels they have been deprived, mistreated, and have grievances directed at a system that they perceive as unjust (33–35). While CHWs in multiple countries express individual grievances with their working conditions and the public health conditions of their communities (1, 11–13, 18, 36), there is less research on how CHWs identify that they share common grievances or experiences with other CHWs based on their collective role as CHWs (37, 38). Thus, the module aims to expose CHWs to the history and background of CHW programs worldwide, explain the unique role that CHWs play in achieving universal health care globally, and share ideas for making professional CHWs a norm worldwide. This module also introduces CHWs to different stakeholders in healthcare and requires CHWs to reflect on working with (1) Government, (2) Multilateral institutions, (3) Donors, (4) Non-governmental Organizations and (5) Civil society, respectively.

**Figure 1:**
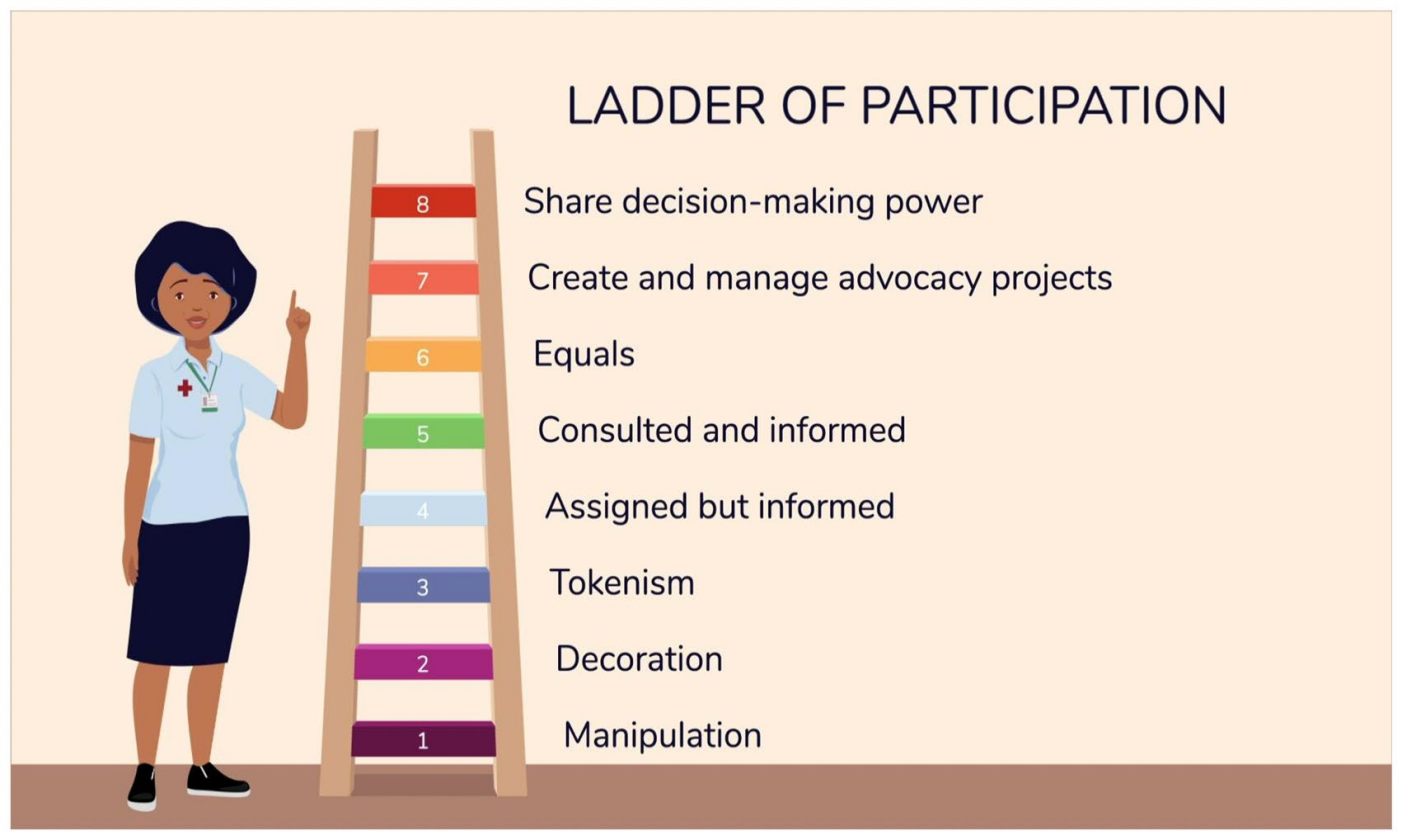
Course infographic: ladder of participation.

The second module focuses on defining advocacy in the context of community health, providing a framework for developing an advocacy project, and exploring how to build coalitions. The module teaches CHWs core advocacy skills in seven (7) steps, with steps 2 and 3 encouraging CHWs to consider both their aspirations and the limitations they may encounter, enabling them to make informed decisions and take effective actions toward their goals. The module uses the symbol of a ladder of participation as a tool for explaining the different levels of participatory decision-making power. Adapted from Hart’s participation from tokenism to citizenship model (39), the ladder of participation presented in the CHW Advocacy Training course has eight levels, with the lowest/first level being ‘manipulation’ and the highest level being a ‘decision-maker’, as can be seen in Figure 1.

The third module of the course focuses on storytelling and is included to train CHWs on how to communicate their own stories to advocate for change. Digital storytelling for CHW advocacy has frequently been used in previous CHW advocacy training, healthcare advocacy training, and community organizing (40–42).

The final module focuses on providing the CHW participants with training on how to use technology tools to participate in virtual global discussions about community health. Research has found that CHWs need ongoing training to use new digital tools (15, 43–47).

## Materials and methods

### Research design

This study employs a design-based research approach to explore the influence of participation in course development on CHW perceptions and how CHW participation shaped course development. DBR is a research methodology that involves research subjects as key collaborators in the research through their contribution to the formulation of the research, allowing participants and researchers to work together on the projects designed, initiated, and managed by researchers (25–31, 48–50). This method allows for the co-development of interventions, adaptations, and iterations on course content, design, and the delivery method of the intervention while supporting the research team to explore the CHWs’ perspectives on advocacy, leadership, and their professional roles. This approach allows for a better understanding of how and why interventions may or may not work in practice and therefore allows researchers to iterate on designs and content to increase the validity, objectivity, and relevance of interventions in real-world educational settings (25–31, 49, 50).

By using a DBR approach, researchers are able to design a curriculum based on principles derived from prior research with the support of research subjects who are collaborators in the research process, i.e., helping to formulate the problems, refining designs, evaluating the effects of interventions and reporting the results of the interventions (26, 28, 30, 31). The iterative nature of this approach draws on a variety of qualitative and quantitative research methods to refine and rework the intervention based on the context. While previous research has indicated that 73% of the studies using the DBR approach were conducted in the United States (25), our study seeks to apply this approach to a global context. Recognizing the need for different methodological approaches to studying educational practice in healthcare, researchers have applied the DBR framework in professional health education research (31) as it has been an effective and innovative research methodology, especially when applied to educational interventions for the purpose of practice change.

Since DBR is concerned with the relevance and contextual usability of interventions that are grounded in local practice (26, 27, 29), 25 CHWs from low-and middle-income countries were identified by CHIC and invited to participate as an advisory group in a series of workshops (four during the course development and one end line) to discuss, evaluate, and refine course content. The CHW Advocacy Training course, described in the Background section, consists of four modules: (1) background on community health systems; (2) advocacy skills; (3) storytelling; and (4) use of technology and tools. The workshop facilitators used semi-structured open-ended questions, informal communication, and cognitive interviewing techniques to guide discussions.

### Participants

CHIC staff identified a purposive sample of 25 CHWs from the Coalition to participate as a CHW advisory group to contribute to course development and refinement. CHWs were selected based on their availability and record of community service. The 25 CHWs are from eight countries: Guatemala, Kenya, Liberia, Malawi, Nepal, Sierra Leone, South Africa, and Uganda. Participants represent varying CHW roles, years of experience, and levels of supervisory responsibility.^2^

All participants in the advisory group are fluent in English, over 18 years of age, and have the capacity to give consent. Over half (56.3%) identify as female. The CHWs range between 30 and 40 years of age. Half of CHW participants (50%) have completed at least secondary education and 43.8% have completed some tertiary education. Almost half of the participants (43.8%) have worked as CHWs for 1 to 4 years, and nearly half (43.8%) have worked in community health for 9 to 16 years. Almost all of the participants are responsible for supervising other CHWs, with the majority supervising fewer than 30 CHWs. The participants mainly serve rural communities (85.7%). A quarter of the CHWs care for between 100 and 150 patients, and over half of the CHWs (58.3%) care for 50 or fewer patients. The CHWs’ work areas are focused on many health topics, with most indicating that they work in maternal and child health and general health education. They also provide services in fields such as hygiene and sanitation, family responsibility, and gender-based violence.

### Data collection

The research team engaged the group of 25 CHWs to provide iterative advisory input over a six-month period (between December 2020 to May 2021). The CHWs were invited to participate in a series of four workshops held virtually via Zoom. Each focus group workshop was two hours long and held at four to five-week intervals. Given that the target audience for the course is CHWs living and working in low-and middle-income countries, the advisory group consisted of current CHWs actively working in community health.

**Figure 2:**
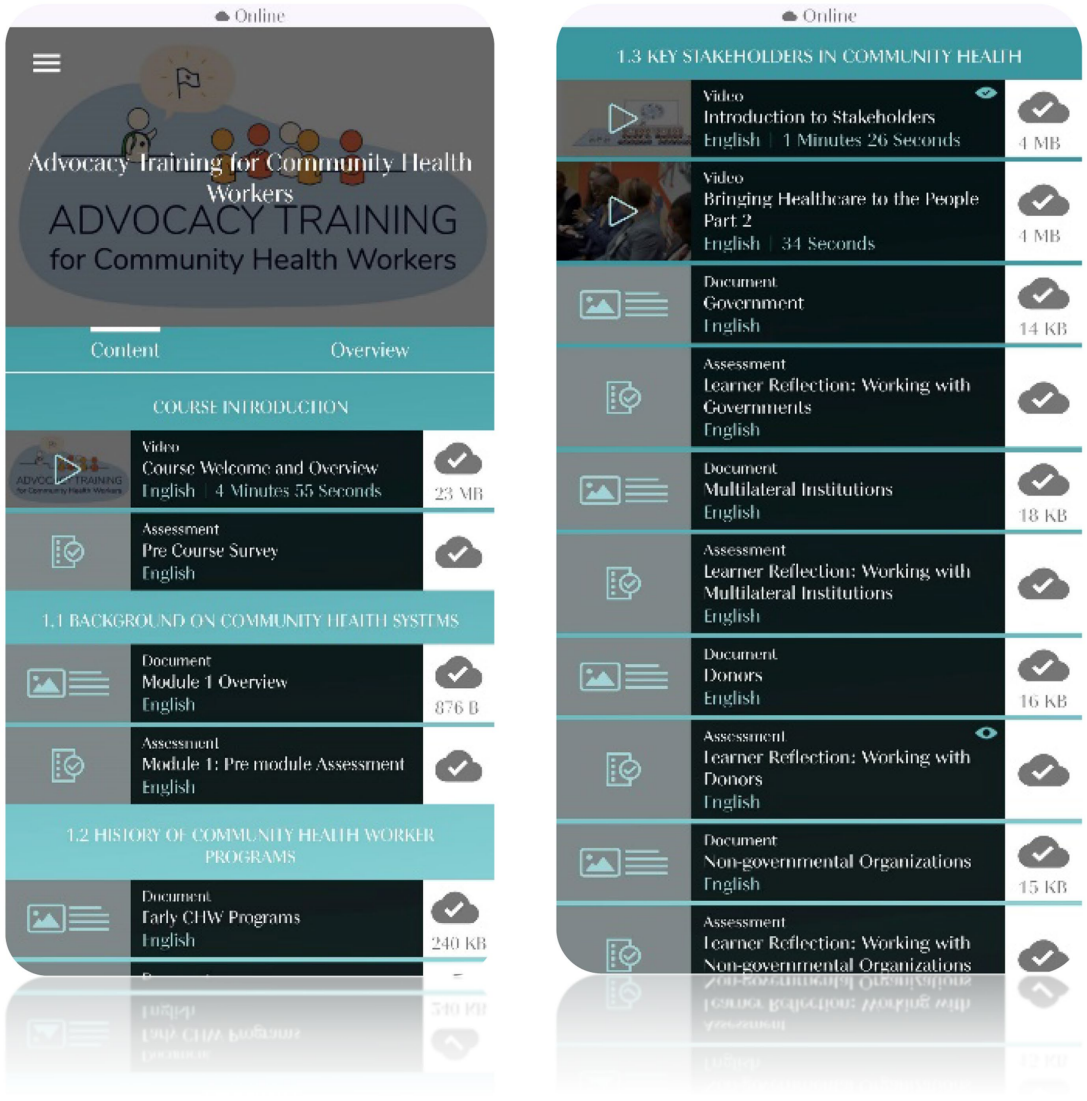
Screenshots of mobile application course interface.

The focus group workshops were audio-recorded group discussions in which the CHWs helped to drive content by reflecting on their experiences and providing feedback on prototype content and the mobile app. Prototype content was shared either before the workshop via a WhatsApp channel or during the workshop. Discussions were facilitated by research team members using a semi-structured discussion guide, which included different kinds of verbal probes to understand interpretations of the course content and wording, adequacy and understandability of assessment question items, the visual appearance and relatability of images and videos, and to tease out any problems encountered while using the app. Figure 2 shows screenshots of the mobile app’s course presentation.

This study draws upon three primary sources of data from the course development process: (1) four focus group discussions held as part of the CHW advisory group workshops, (2) endline focus group discussions (split into smaller groups based on geographic location) following the last workshop, and (3) open-ended online survey data collected throughout the study period. An open-ended baseline survey (administered prior to the start of the workshop series) and pre/post surveys (requiring roughly 10 min to complete and collected before and after the second, third, and fourth workshops) provide additional context on the background and experience of CHW participants. All surveys were administered digitally using the Qualtrics XM survey tool.

During the workshops, semi-structured interview protocols and cognitive interviewing techniques were employed to test the face validity of the course. The focus group facilitator used different probing techniques to understand CHWs’: (1) interpretation of course content and wording, (2) comprehension of assessment items, (3) resonance with images and videos, and (4) problems encountered while using the mobile app platform. General probes included, for example, “What do you think of this introduction?.” Comprehension or interpretation probes included, “What does this ‘word’ or ‘image’ mean to you?” or “What do you think this ‘image’ would mean to other CHWs in your area?.” Paraphrasing probes included examples such as “In your own words, how would you say this?.” Observational probes included, “Why did you not answer this question?” or “Why did you hesitate to answer that question?.” Content probes included, for example, “Is this ‘item’, ‘image’, or ‘word’ relevant in your context?.” Finally, comfort probes included, “Were you comfortable answering that question?”

Prior to participating in the course development process, the CHW participants completed a baseline survey. The baseline survey asked about individual-level variables, including basic demographic information, education level, previous CHW training, and years of experience in the community health profession. The baseline survey also asked about patient-level variables, including the number of patients served, demographics of patients served, general concerns about patients, and access to technology in their practice.

Table 1 illustrates the attendance (number of respondents) over the six-month period and the content focus for each focus group workshop discussion. Due to scheduling conflicts and limitations with technology access, not all CHWs could participate fully in every course development activity. The workshops focused on content covered in the first three modules, while feedback on technology access and skills were collected via surveys.

**Table 1:** Workshop focus group discussions (FGD) and survey participation.

Date	Content focus	Activity	*N*
December 10, 2020	*Workshop 1: Introduction* Participants were introduced to each other and the course development team. They were provided an overview of course objectives. Participants were asked to reflect on their experiences as CHWs.	Baseline survey	21
		FGD	18
January 26, 2021	*Workshop 2: What is Advocacy?* Participants were introduced to the learning objectives for the first module and previewed prototype content on the 9-step advocacy process. Participants provided feedback on their understanding of advocacy.	Pre-survey	16
		FGD	19
		Post-survey	15
February 17, 2021	*Workshop 3: Ladder of Participation* Participants were introduced to the learning objectives of the second module and previewed prototype content focused on the Ladder of Participation infographic. Participants shared feedback on how to refine the infographic and reflected on their placement on the Ladder of Participation. Participants were also introduced to the course mobile app platform and asked to reflect on challenges associated with accessing and utilizing mobile technology in their work.	Pre-survey	14
		FGD	21
		Post-survey	14
March 25, 2021	*Workshop 4: Storytelling* Participants were introduced to the learning objectives of the third module and prototyped storytelling content, with a focus on creating and sharing personal stories.	Pre-survey	18
		FGD	16
		Post-survey	15
June 2021	*Endline: Regional FGDs* Participants were organized into three smaller focus group discussions, organized by region, and reflected on their experience with the course development. They provided recommendations on how to further refine and distribute the course to CHWs globally.	Region 1 FGD	3
		Region 2 FGD	6
		Region 3 FGD	4
		Total	13

### Data analysis

To analyze the focus group data, a codebook was generated using *a priori* codes from the literature on community organizing and health advocacy. Each focus group transcript was independently coded by two team members (NJ, JSJ, CW, and MB). Open-ended survey responses were also coded independently by two team members. Members of the research team met multiple times to confer and calibrate coding interpretation and to refine further and recalibrate coding schemes to ensure coder reliability. The transcripts of all workshops and open-ended questions from the surveys were analyzed through thematic coding using Dedoose data analysis software. Next, researchers used constant comparative methods to systematically code data and identify the themes emerging from interview data. The team then met and revised the codebook again to include both inductive and deductive codes.

### Ethics approval

Informed consent was obtained from all CHW advisory group participants. Approval for this study was granted by the Stanford University School of Medicine Institutional Review Board (Protocol No. 59174).

## Results

Four primary themes emerged from the analysis. First, CHW participants entered the training with an understanding of health advocacy based on advocating for vulnerable communities and saw themselves as advocates for their communities. Second, while CHWs believed in advocating for their communities, as the training progressed, they struggled to reconcile the idea that advocating for themselves and their own working conditions does not conflict with their care and advocacy on behalf of their patients and communities. Third, as the workshop series progressed, the CHWs began to identify as part of a cohort of professional health workers with a unique role in supporting health systems globally. Fourth, the CHWs embraced their role as advisors, reflecting positively on their own decision-making capacity and influence on the course creation.

### Theme 1: CHWs entered the training with an understanding of the concept of advocacy and clearly perceived themselves as advocates for the health and well-being of their communities

During the first two workshop discussions and in open-ended responses to early surveys, the CHWs were invited to share their pre-existing understanding of advocacy. The CHWs explained their understanding of the term ‘advocacy’ as being a representative for others or speaking on the behalf of others. An example of this type of belief can be seen in one participant’s definition of advocacy. The participant states, “*advocacy means ‘the voice’, the voice of representing a group or some people, a group working together, but you represent them”* (CHW 005, Workshop 1).

The CHWs expressed an understanding of advocacy rooted in the idea that one who advocates is doing so “*on behalf of others”* (CHW 015, Workshop 1). Several CHWs specifically described *“others”* as members of their communities who are *“voiceless,”* sharing that their task as CHW advocates was *“giving a voice to the voiceless”* (CHW 022, Workshop 1) who are too vulnerable to speak for themselves. One CHW’s definition of advocacy provides an example of this idea, as their definition is grounded in the idea of speaking for vulnerable peoples. This CHW states “*advocacy means to speak for those that do not have the means and power to speak for themselves”* (CHW 022, Workshop 2 Pre-Survey).

Related to the idea that advocacy should be on behalf of others, the CHWs expressed that advocates can solve problems because of their vast knowledge of socio-political systems and networks within their communities. These explanations of advocacy work, particularly shared during the second workshop discussion and surveys, were often laden with ideas that advocates should be knowledgeable about bureaucratic structures, be able to build rapport within different socio-political structures, and teach people their rights and make them aware of opportunities available to them. The CHW participants indicated that the minimum role of advocacy was education because advocates have knowledge. This role of an advocate is expressed by a CHW participant who stated, *“to advocate is at least to educate the community. The CHWs are advocates”* (CHW 001, Workshop 1). In addition to education, many CHWs also indicated that the knowledge that advocates had should be used for supporting those who did not have the same access to knowledge. This role of advocacy is expressed by a CHW participant who stated:

*“Advocacy is to talk on behalf of somebody who does not have the power to plead on behalf of themselves, representing somebody who is not respected, and who does not even know they have rights, and you know that they have rights, who does not even know that maybe they are entitled to opportunities. So, because of that, you have some of us who talk on behalf of those people, to let them know that these people have rights, to plead on behalf of them so that some opportunities can be given to them”* (CHW 015, Workshop 1).

The CHWs saw themselves as ideal advocates for their communities and well-positioned for the work of advocacy, given their role as health providers. The CHW participants came into the workshops with definitions of advocates being likened to *“bridges”* (CHW 019, Workshop 2) between socio-political structures and the community, describing CHWs as *“the bridge”* between the healthcare system and the community. The CHW participants expressed that in the healthcare field their breadth of knowledge across health topics, including mental health and stigmatized health topics, such as contraception and HIV/AIDS, positioned them as better health advocates. They consider themselves to be well-positioned to speak on behalf of their patients when reporting to the health facilities because they are able to fully understand the problems that emerge in their communities and the need for real solutions. An example of this conviction of the value of their breadth of knowledge for community health advocacy can be seen from one participant who stated:

*“It would be better for CHWs to be advocates because they are the ones who are staying in the communities and they know people better than any other person because they are the ones who are there… They know their day-to-day livelihood so they can speak out because they know what they are saying”* (CHW 002, Workshop 2).

In the surveys administered prior to the second workshop discussion group, many CHWs also expressed that they viewed advocates as mediators between the government and local people and communities. They expressed that as community advocates they believed that their roles as CHWs were to serve as liaisons between the community and community structures. This liaison role included structures that were concerned with maternal and child health but also with sanitation and hygiene, safe drinking water, women’s rights, children’s rights, youth health, and gender-based violence.

### Theme 2: CHWs experience a moral conflict between advocating for themselves and their working conditions and empathy for their patients and employer organizations

The CHWs were well aware of and could articulate the problems associated with being a CHW. They provided many examples of challenges in their line of work that they believed were hindering them from working optimally. Some of the challenges they discussed included: the lack of transport for getting to hard-to-reach-areas; stigma and discrimination from the community; lack of personal protective equipment and other needed gear (e.g., gumboots and raincoats in the rainy season); lack of work equipment (e.g., scales and other resources); high CHW to household ratios; lack of training and support; and late and/or lack of adequate remuneration. Often, as a result of these challenges, the CHWs expressed that they could not meet the expectations of their patients, who needed more health services, supplies, and information. One CHW shared:

*“We are in the community, working as CHWs. You find that some people are not recognizing us as health workers… CHWs were given this [personal protective gear] PPE very late, some have not even received the PPE…”* (CHW 014, Workshop 1).

The CHWs also articulated challenges they encountered while working with other healthcare staff in health facilities. They indicated that they were not recognized as fellow health workers since *“it’s a work of no promotion. We belong to the community. They [other health workers] feel we are not competent because we talk of health issues and they think we are not trained”* (CHW 017, Baseline Survey). Thus, the CHW participants expressed that they dreaded engaging with other health workers. They often expressed they did not work collaboratively with other health workers whom they believed considered the CHWs to be lay personnel without real qualifications. The CHWs argued that their role in referring patients, who would have otherwise stayed home, to facilities was important. Nevertheless, they stated that their referrals were sometimes undermined and their patients were not assisted.

At the start of the course, the CHW participants were not confident about presenting these issues about their welfare to their superiors. The discomfort with approaching their superiors can be seen in this comment by a CHW who stated, *“we have talked about it [referring to late/no remuneration, PPE and not being recognized as health workers] with our leaders. In some parts we are comfortable and in some parts we are not comfortable”* (CHW 014, Workshop 1). However, they believed that their governments’ leadership should assist the CHWs with their challenges. This belief seemed to increase during the course. They expressed a general idea that governments were expected to ensure the well-being of CHWs because the work they do is necessary and visible to the government. They were confident that their governments were aware of their efforts but acknowledged that they were not prioritizing the formalization of the CHWs’ work or the role they play in their communities. This idea can be seen by one CHW who stated, *“this work we do, it is evolving, even the government is aware… they see. All we need is a matter of time, to come together, to solve this problem together”* (CHW 005, Workshop 2). Another CHW shared this idea about lack of acknowledgment of their work from the government stating that the *“community needs us, but the government does not recognize us, being they do not pay CHWs”* (CHW 024, Baseline survey).

However, even with their recognition of the challenges they faced as CHWs, during the first two workshops and earlier surveys, the CHW participants rarely mentioned advocating for their own agency or engaging in ‘workplace advocacy’. Advocacy discussions focused on advocating for their community and doing work that will serve the entire community. When the course introduced the concept of ‘workplace advocacy’ during the first and second workshops, the prominent response among CHWs was that one should not advocate for oneself because one’s intentions may be perceived as self-centered. They shared their impression that if they continued to do good work in their community, it would be recognized and eventually rewarded, without a need to advocate for rewards, remuneration, or recognition explicitly. An example of this belief can be seen in this quote from a CHW participant who stated during the fourth workshop discussion group:

*“You take an action that would benefit the whole community, not only the health community worker. Because it will be translated in other ways that maybe you are fighting for this, just for your own benefit but if you are making such for the benefit of the community, your own benefits will come automatically”* (CHW 016, Workshop 4).

The CHW participants fully acknowledged that they require remuneration, and increased and timely pay, to sustain their families. Nevertheless, they began the workshops reluctant to speak on money-related issues. As the workshops progressed, they expressed that part of their initial reluctance to discuss remuneration for their position was because, by being CHWs, they were well-intentioned and they *“love their communities”* and their jobs, which they felt was in conflict with advocating for remuneration. When directly asked about this idea by researchers, a few CHWs shared their opinion. One CHW shared:

*“We volunteered and we are for our communities. We’ve done it for so long and we really love to do it. But, when we are doing the work, or even when you are walking in the community, walking door to door, there should be something that can sustain your family…of course there’s shyness there. There is… We cannot open up to talk about money because we are volunteers”* (CHW 009, Endline FGD).

Beyond the CHWs’ reluctance to speak about money for fear of being misunderstood as ill-intentioned, they were reluctant to mention remuneration because they had accepted the role of CHWs to ‘volunteer’ for their communities. They reasoned that it would be inappropriate to complain when they had been made aware that they would not receive an income. One CHW presented this reluctance as follows:

*“We took on the job knowing that we are not there to, like, we are not paid much so we have empathy. That empathy is directly for those people that we care for. So, because of our empathy that we have for these patients and other people in the community, we are there to advocate for them”* (CHW 015, Workshop 2).

The CHWs also expressed a deep sense of understanding, or empathy, for the organizations that they work for, which they accepted as limitations for the support they received. They perceive that their employer organizations need more funds to pay them. They have observed their organizations run out of resources and often ration the little they have among their communities. One CHW shared:

*“The organization does not have enough money to support the project. Rather, we proposed … [to be] provided with motor bikes so that that can be extracted from our earnings but the organization said that they do not have much money at least to try out that”* (CHW 001, Workshop 1).

Likewise, another CHW shared:

*“The government also relies on [NAME OF EMPLOYER ORGANIZATION]. So, if [NAME OF EMPLOYER ORGANIZATION] does not have money, then the government does not have money. So, it is very difficult… When our bosses say that we do not have money, we are just waiting, maybe… the money will be there… but currently, it is very, very difficult…”* (CHW 002, Workshop 1).

### Theme 3: CHWs began to identify as part of a global cohort of professional health workers and recognize their ability to uniquely advocate for themselves and their communities

In workshops 3 and 4, new themes started to emerge during discussions. CHWs began to express a sense of identity with the CHW field and confidence to advocate for their position. They expressed appreciation for the module on the history of the CHW profession. They indicated it helped validate their work and positioned them in some rank of importance in the health system. This validation is exemplified by a CHW who spoke about the value of learning about the history of their roles, and said, *“the history [of CHW programs], the value that you give the people, it’s going to help every CHW to overcome that shyness, so that we can advocate ourselves in the community”* (CHW 012, Endline FGD). Another CHW agreed, sharing, *“the reading [referring to the module on the history of CHWs] is just so good because they’ll pave a way to the CHWs and where they are coming from*” (CHW 001, Workshop 3). The CHWs expressed that the module was important in helping them justify their work and reassuring them that they belonged to a greater, global community of professional CHWs. This helped assert that they are doing important work, regardless of what others who did not recognize them as other health workers may have said previously.

The CHWs indicated that the course would be valuable for training all CHWs because they thought it would give them identity and better clarify their roles and responsibilities as CHWs and fellow health workers. One CHW participant explained their reasoning for wanting more CHWs to take the course by stating:

*“before the course… most of the things were hidden to us. We did not know our roles, what we should be doing. [When other CHWs take the course], they will now understand their roles, they will now understand what they should be advocating for, through this course that we came up with”* (CHW 017, Endline FGD).

The CHW participants expressed that they found value in interacting with the other members of the advisory group. The interactions helped demonstrate that they belonged to a greater, global community of professional CHWs. The CHW participants interacted with each other both during the workshop discussion groups and on a WhatsApp channel created for the group. In these interactions, they discussed the challenges they faced as CHWs and they often found them to be similar. They shared that this experience validated their knowledge and roles, as they realized that CHWs existed in other countries and experienced the same challenges in their workplaces. One CHW shared:

*“I’ve realized we, as CHWs, our voices can be heard and we can change our community… We have met with our colleagues from other countries, and we have found that the work that we are doing in these rural areas are just the same as others”* (CHW 009, Endline FGD).

As the course progressed, the CHW participants increasingly reflected on the merits of advocating for better working conditions. This advocacy is exemplified by this statement from a CHW where they said:

*“CHWs themselves should advocate for their stipend after work. They also should advocate dually for the CHWs to know and feel that they have the knowledge to be giving to the community members that can change the environment where they are tasked.”* (CHW 017, Endline FGD).

The CHW participants indicated that they could not rely on their good works to “speak for them” but started suggesting that they were responsible for advocating for their well-being and recognition in the workplace environment. They began to suggest that they were the ideal advocates for their well-being in the same manner that they advocated for their patients. One example is a CHW that said, *“okay, so far I can say that the CHWs themselves should advocate for themselves”* (CHW 009, Endline FGD).

CHWs identified stories as essential tools for communicating with their patients and that telling their own stories to their patients improved trust and facilitated change. One CHW shared*, “you are telling your story, you have a story that will catch people. And when you come to clients, you do not give up because one time, one day you will come for a change as a community even as individuals”* (CHW 014, Workshop 4). They also indicated that they could use their stories as promising tools for advocating for themselves as CHW 014 continued to share:

*“In our storytelling also, we have to know that we are having so many challenges in the field when you are talking to people so you have to speak out, not to be afraid anymore. And if you are speaking out, you will help the community and your fellow members and CHWs”* (CHW 014, Workshop 4).

They expressed an appreciation for how the course gave clear steps to effectively tell stories and realized that they could use the method for their own agency.

During deliberations about the importance of their work and the little to no incentives to keep doing it, they reflected on the lack of pay and, in certain instances, the late payment of stipends, and how negatively this affects their lives. They also acknowledged that they should advocate for themselves concerning improving their training and qualifications, resources required to conduct their work, PPE, and means of transportation to distant patients. One CHW participant shared:

*“The first people who should advocate for the welfare of our CHWs, is ourselves, because what I know is that we cannot work for somebody to fight for us. Because we are the ones working on the ground. We are suffering. We are meeting a lot of challenges. We are there to fight for our friends. We are there to fight for ourselves, to live a better life, to work in a good environment… decisions are just made without involving [CHWs]… so we need to fight for that… we are working the field. We are working in the community as well. We are not given necessary materials to work. For example, I thought of traveling long distances on foot over 5 kilometers walking… We need to fight for that. Government should listen to us, provide us with good resources, good PPEs… those are some of the issues we need to fight for… we need to advocate for that”* (CHW 016, Endline FGD).

Some CHW participants began to express that by self-advocating, they could support their communities better. This can be seen by a participant who stated:

*“The CHW advocating for their own good… financially, socially and maybe also healthwise, because they are there to help other people but their own good is not like… They are in the frontline helping other people when they are not helped themselves… they should first advocate for their own health so that they will be in a better place whenever they are advocating for other people”* (CHW 002, Endline FGD).

The CHW participants found the ladder of participation presented in the training to be a valuable tool to measure themselves against and expressed that it was illuminating for them. When first presented with the ladder of participation, the CHW participants identified themselves and their fellow CHWs as occupying the lowest levels, in the bottom three tiers, of participatory decision-making. An example of this can be seen from one CHW who shared:

*“In most of our countries, our places of work, as CHWs, most of us are in the manipulation stage, because you find that some that are working with us, all they want from you is to be walking up and down without you having any voice to say… as a CHW I should be climbing up the ladder… Actually at the moment, the CHWs are just in the manipulation part, decoration, and tokenism. Those three steps are where we are at the moment”* (CHW 011, Workshop 3).

Some CHW supervisors, while recognizing that they occupy low levels of decision-making participation, acknowledge that the CHWs they supervise, those working at the village level, occupy levels that were lower than theirs, the very lowest levels of participatory decision-making of manipulation, decoration, and tokenism. This can be seen from one CHW who stated:

*“The CHWs are doing work on the ground, the decision-makers are not aware of what the CHWs are doing on the ground… these CHWs are the ones that are doing good work on the ground, but they are now on the first stand-manipulation… they are just being informed… they do not have power”* (CHW 001, Workshop 3).

They reflected on how their voices were often not heard or taken seriously by those with decision-making power. They argued that their voices should be more recognized than they currently are as they work with their communities directly and have valuable contributions to make. They even associated inevitable program failures with the inability of leaders to listen to CHWs’ voices and include them in decision-making. For example, one CHW shared:

*“The guidelines come from the top to the bottom. Who knows the realities of the communities? It’s the CHWs! This is a big issue here and sometimes the guidelines do not work”* (CHW 010, Workshop 4).

Another CHW participant stated:

“*We are voiceless…We are unrecognized. Only when they want to make use of us, we are recognized because decision making we are not part of. We only implement. We implement after making their decision, we implement. That’s why sometimes they miss the goal, because they aren’t including us in decision making”* (CHW 022, Endline FGD).

They perceived that they were considered unimportant since they are not invited to make decisions. Many CHWs shared that they were only invited to implement programs after leaders had made their decisions. Several CHWs also indicated that when they were invited to meetings, they were mainly invited to listen and learn, not participate in any other way. One CHW participant shared, *“you are invited and you are maybe there to sit… watching them talk, you only listen and you are not given any chance to talk. You are there to take notes… You are there to increase participants”* (CHW 005, Endline FGD).

After reflecting on the low levels of participation they occupy in their organizations or facilities, the CHWs were optimistic about reaching level/tier number six presented in the model, which is ‘being equals’ with their fellow health workers. While the module aims to enable each participant to see themselves as able to reach level eight and aim to influence decisions in their place of work, the CHWs were more hesitant about reaching this level. Many participants indicated that it was most important for them to be seen as equals by others, especially by other health workers, and were therefore interested in reaching level six.

They expressed hesitancy about striving to reach the highest level of the ladder of participation – decision-making (i.e., ‘level 8’ as referred to by CHWs). When addressing the possible causes of this hesitancy, one participant shared:

*“My dreams are to reach level eight… we have got that potential of reaching that far, maybe it’s just because there are other issues that are associated with CHWs, things like oppression, things like not being recognized. But, as we have taken this course of advocacy, maybe things will change, we will reach that level… Knowledge is power. Starting with me and the other CHWs after all the courses, after knowing who is who, who to consult, all the stakeholders… we can reach level 8… we are aiming at nothing less”* (CHW 013, Endline FGD).

Many of the participants indicated that they saw moving up the ladder as a process and believed that they could aim for higher levels after attaining inclusion in lower participation levels of the ladder. An example of this idea can be seen from one CHW, who shared:

*“Okay, for the CHWs, I think it cannot be possible for them to reach ladder number 8 without reaching number 4, 5, 6, going upwards…. We want to be equals. If maybe our colleagues are doing this, we should be able to do the same”* (CHW 002, Endline FGD).

Another CHW shared this idea about the possibility of growth, stating:

*“Since we started [the course] we can now see that at least we cannot be just there at the bottom. We can be raising, and we can also go up to number 8. I think every community health worker is just like me, I feel now there’s nothing impossible. We can be equals, we can be consulted, we need to share in decision making. I think we are growing as CHWs and now we can see the light at the end of the tunnel”* (CHW 005, Workshop 3).

### Theme 4: the CHWs embraced their role as advisors and reflected positively on their decision-making influence in the course creation

All of the CHW participants reported that this was the first time they had been invited to participate in developing a course. Some reported being uncertain and reluctant to give critiques or comments until they realized that their recommendations and suggestions resulted in content changes that were shared with them in the following workshops. They reflected that they were often invited to meetings, but their suggestions were never implemented. Seeing the incorporation of their recommendations into the course design gave them the confidence to comment more freely on the content and how it would resonate with their fellow community health workers from their respective countries. With increased interaction with the course and the different elements of the course, the CHWs provided more direct and detailed feedback. As the CHWs observed the impact of their participation on the course development they experienced a sense of ownership of the course and stated that they would be confident enough to take the course and train their fellow CHWs on all the information given. The CHWs expressed that they were heard in the development of the course. As one CHW shared:

*“According to what I was seeing, my views were heard because I was able to see some changes whenever we say, ‘this maybe should be changing to this’, ‘maybe we should edit this’. In the next meeting I would observe that there were some changes! So, I would say, yes my views were heard and it was helpful so that the course will be as it is now”* (CHW 002, Endline FGD).

Table 2 displays a summary of the types of course development suggestions from CHWs recorded by [*Name of Institution*]‘s instructional design team throughout the workshop series, along with a description of how the instructional design team revised the prototype content. Our analysis of the log of CHW suggestions shows that the CHWs largely embraced the course learning objectives and believed the course content was relevant and valuable for CHWs like themselves. The CHWs gave specific suggestions for the presentation of the material to improve course accessibility for non-native English speakers and to improve resonance across contexts. The CHWs also provided feedback on how the course could be optimized for access for limited technology options and bandwidth restrictions.

**Table 2:** Summary of course development suggestions from CHWs.

Typology	Suggested changes	Changes made
Length, sequencing, etc.	Reduce video size to accommodate low phone storage and data consumption	Majority of the 43 videos capped at 11 MB, with a median of 11 MB, mean of 12.5 MB, min of 2 MB and max of 56 MB
Wording, language	Use simple English Add definitions to key words being used in the module. For example, key steps in the ladder of participation Edit confusing or difficult multiple choice questions Clarification of instruction on affirmation exercise Translate course into local languages	Advanced English wording changed throughout course Puppet narrator gives examples of each stage in the ladder of participation in a video. Problematic words and response options changed in assessments throughout course Instruction on affirmation exercise changed Course translated into French and Spanish
Image depictions	Dress code edits to narrating puppet to match most common CHW dress code Addition of friendly gestures to narrator to reflect warm personality of a CHW Include an additional male narrator for gender representation More video requests to accommodate those who cannot read More contextual images of surroundings. For example, change of buildings to reflect villages not cities. More relatable image representations (relatable nuances). For example, image of winding road map to demonstrate the journey of a plan not well understood	Puppet dress details changed to include both pants and skirt, ID badge and updates to how the backpack is worn Friendly facial and hand gestures added to narrator All key topics explained in video format Images of buildings and surroundings changed Roadmap illustration changed to a clear upward moving roadmap
App technicalities	Data issues: trade voice overs for text only to reduce file size Network coverage and data costs make download difficult Those who cannot read and write will have a challenge installing the app	More detailed texts added to course without voice over Majority of file sizes for download capped at 11 MB Facilitator and learner guide developed for in-person or hybrid training How to install app guide created Creation of progressive web app version of the course at www.chwadvocates.app

## Discussion

At the beginning of the study, the CHWs clearly perceived themselves as advocates for the communities they serve but not necessarily as advocates for their profession—despite the majority of CHWs being unsalaried and CHWs being out of stock of essential medicines 1/3 of the time (52, 53). As the CHWs engaged with training content, they began to identify as a global profession of CHWs and see the value in advocating for themselves. Their understanding of workplace advocacy evolved from a moral conflict to a way to support their community. By the end of the process, CHWs reflected positively on the impact of their role in shaping the course and experienced a sense of ownership.

The 25 CHW participants entered the course design workshops with a clear definition of health advocacy that recognized the role of community health workers as advocates for vulnerable communities. The CHWs acknowledged that they performed a wide range of advocacy activities for diverse issues that emerge in their communities. Their preconceived notion of advocacy heavily entailed the idea that CHWs should understand bureaucratic structures to foster relationships across socio-political contexts and educate individuals about their rights and opportunities. We conjecture that this idea that advocacy requires a detailed grasp of health systems bureaucracy could function as a barrier to entry, limiting CHW involvement in the advocacy space. Additional stakeholders, such as government officers, can play an important role in assisting CHWs with navigating bureaucratic structures and better incorporating them as advocates more largely for the improvement of health outcomes across communities.

The CHWs also entered the workshops acknowledging the challenges they face related to their working conditions. They recognized the impact of poor working conditions on their well-being and their patients’ well-being. Among the challenges, the CHW participants indicated that they feel unrecognized as health workers by other health workers and receive a lack of support from their employer organizations and partners, including, in some cases, the government. The participants reflected that commonly CHWs are unpaid, inadequately trained, and rarely invited to participate in decision-making – all factors contributing to feelings of unimportance.

Despite clearly recognizing the challenges they faced, as the workshops began, many CHW participants were uncomfortable with advocating for themselves. The CHWs mainly felt conflicted about advocating for better pay for themselves. One of the primary barriers to embracing self-advocacy was their perceived moral conflict between doing passion-driven, voluntary work for their community with actions aimed at improving their situation. They expressed that by advocating for themselves, resources may be allocated to them at the expense of their communities. Other barriers to self-advocacy included their lack of confidence over what they perceived as their insufficient training or formal education.

As the course design workshops progressed, many CHW participants began to see themselves as part of a global profession of CHWs. They shared their aspirations for greater CHW involvement in decision-making. This helped them identify the challenges they faced in a global context. Discussions about the levels of participatory community health decision-making they experienced as CHWs helped solidify for many CHW participants a need to become more active as self-advocates. The CHWs acknowledged that their work as CHWs would not automatically lead to greater recognition by those in power and that self-advocacy is important to promote change.

Overall, the CHWs reflected positively on their contribution to the refinement of the course and the iterative feedback provided during the workshop series. Some participants expressed that they were initially reluctant to provide recommendations and grew in confidence to share feedback when they observed prior suggestions were incorporated into the course. At the same time, the CHWs recommendations were largely focused on the presentation of materials to improve resonance with the CHW audience. We acknowledge that involving CHWs in the earlier stages of curriculum planning and establishment of learning objectives could elevate their role in course development.

Limitations in the present study allow for future work to investigate how advocacy training can prepare CHWs to support their communities better and advocate for themselves at scale. First, as this is a relatively small cohort of CHWs specially selected to participate in the co-creation of the training, these results cannot be generalized to the entire population of CHWs. We recognize that the applicability of the course content will vary across contexts globally. We recommend that future studies investigate ways to tailor course modules and recommended practices to better equip CHWs as advocates across vastly diverse health systems worldwide. Second, the CHWs interacted with the others in the advisory group as part of the DBR approach. Further research will need to determine which modality components best support the development of a connection between CHWs from different countries and regions and if this can be replicated without facilitated interactions. Finally, future studies should incorporate experiential components of digital training for CHW advocacy training, incorporating stakeholders and training of stakeholders.

## Conclusion

Our study finds that CHWs embrace their role as advocates for their community but encounter challenges that prevent them from advocating on their own behalf, allowing them to support their communities from a strengthened position of being salaried, skilled, supervised, and supplied. Our findings suggest that efforts to engage CHWs in advocacy must overcome systemic barriers and norms internalized by CHWs – particularly, a moral conflict with self-advocacy. Ultimately, exposure to advocacy principles and involvement as co-creators in developing the advocacy training content for fellow CHWs increased awareness of the role CHWs play in health systems, improved the confidence of many of the CHW participants to self-advocate, and enhanced the sense of connectedness between CHWs from different countries and regions. Our study also contributes to the evidence that the DBR approach can facilitate interdisciplinary collaborations between researchers, curriculum developers, and practitioners to create educational tools that can be useful in practice. Future studies should explore how to engage CHWs in advocacy training at scale, allowing them to reach their full potential as advocates.

## Data availability statement

The original contributions presented in the study are included in the article/supplementary material, further inquiries can be directed to the corresponding author.

## Ethics statement

The studies involving humans were approved by Stanford University School of Medicine Institutional Review Board (Protocol No. 59174). The studies were conducted in accordance with the local legislation and institutional requirements. The participants provided their written informed consent to participate in this study.

## Author contributions

NJ: Writing – review & editing, Writing – original draft, Methodology, Investigation, Formal analysis, Data curation, Conceptualization. JJ: Writing – review & editing, Supervision, Methodology, Investigation, Funding acquisition, Formal analysis, Data curation, Conceptualization. CW: Writing – review & editing, Validation, Project administration, Funding acquisition, Formal analysis, Conceptualization. NS: Writing – review & editing, Validation. VW: Writing – review & editing, Supervision, Project administration, Methodology, Investigation, Funding acquisition, Formal analysis, Conceptualization. MB: Writing – review & editing, Resources, Methodology, Investigation, Funding acquisition, Formal analysis, Data curation, Conceptualization.

## CHW Advisory Group

The CHW Advisory Group is made up of 25 CHWs from different member organisations of the Community Health Impact Coalition. The CHWs are from eight countries: Guatemala, Kenya, Liberia, Malawi, Nepal, Sierra Leone, South Africa, and Uganda. Participants represent varying CHW roles, years of experience, and levels of supervisory responsibility.

## Footnotes

^1^ http://chwadvocates.app

^2^The qualifications, job descriptions, and titles for CHWs vary significantly by country (e.g., Health Surveillance Assistant, Village Health Worker, Community Health Volunteer, Community Health Nurse Supervisor, etc.) (51). For purposes of this study, the terms ‘CHW’ and ‘CHW Supervisor’ are used to capture these title permutations.
